# Aggregation Induced Emission and Nonlinear Optical Properties of an Intramolecular Charge-Transfer Compound

**DOI:** 10.3390/ma14081909

**Published:** 2021-04-11

**Authors:** Songhua Chen, Rui Luo, Xinyue Li, Meiyun He, Shanshan Fu, Jialiang Xu

**Affiliations:** 1 College of Chemistry and Material Science, Longyan University, Longyan 364012, China; songhua@iccas.ac.cn (S.C.); meiyunhmy@163.com (M.H.); shanshanFu33@163.com (S.F.); 2 School of Materials Science and Engineering, National Institute for Advanced Materials, Nankai University, Tongyan Road 38, Tianjin 300350, China; 1813791@mail.nankai.edu.cn (R.L.); xinyueli@yeah.net (X.L.)

**Keywords:** intramolecular charge transfer, aggregation induced emission, nonlinear optics, benzothiadiazole

## Abstract

Intramolecular charge transfer (ICT) compounds have attracted wide attention for their potential applications in optoelectronic materials and devices such as fluorescent sensors, dye-sensitized solar cells, organic light emitting diodes and nonlinear optics. In this work, we have synthesized a new ICT compound, dimethyl-[4-(7-nitro-benzo[1,2,5]thiadiazol-4-yl)-phenyl]-amine (BTN), and have fabricated it into low dimensional micro/nano structures with well-defined morphologies. These self-assembled nanostructures exhibit high efficiency solid state fluorescence via an aggregation induced emission mechanism, which overcomes the defect of fluorescence quenching caused by aggregation in the solid state of traditional luminescent materials. We also explored and studied the nonlinear optical properties of this material through the Z-scan method, and found that this material exhibits large third-order nonlinear absorption and refraction coefficients, which promises applications of the materials in the fields of nonlinear optics and optoelectronics.

## 1. Introduction

When conventional organic fluorescent materials engage in π -stacking, the luminescence is partially or completely quenched [1]. Such aggregation leads to aggregation-caused quenching (ACQ), mainly due to the strong π-stacking among molecules and the formation of excimer complexes. Aggregation induced emission (AIE), an intriguing phenomenon, was first discovered and proposed by Ben Zhong Tang in 2001 [2]. AIE molecules, in contrast to conventional dyes, can emit light efficiently in the solid state, which fundamentally solves the problem of ACQ and makes fluorescent materials rich, extended and developed [3]. At the same time, many mechanisms, such as the essential structure flattening, restriction of intramolecular motions (RIM), restriction of intramolecular vibrations (RIV), restriction of intramolecular rotations (RIR), twisted intramolecular charge transfer (TICT), *J*-aggregation formation (JAF), excited state intramolecular proton transfer (ESIPT), excited state intramolecular charge transfer (ESICT) and other theories [2,4,5,6] have been proposed as the explanation of this phenomenon. After nearly 20 years of rapid development, AIE has been applied in various areas of research and is currently an extensively studied field [7]. Many AIE materials have been designed and synthesized for wide applications in the fields such as organic light-emitting diodes, sensing and biological imaging [8,9]. However, most of the developed AIE materials are of short absorption and emission wavelengths, limiting their further developments and applications. In recent years, due to the characteristics of high absorption intensity, narrow emission crest and easy modification, small organic molecules featuring electron Donor (D)-Acceptor (A) characteristics have been applied in near-infrared luminescent materials and devices. In our previous work, it was found that the intramolecular charge transfer compounds were assembled into ordered aggregations with a well-defined morphology by the non-covalent weak interactions at the supramolecular level, which could overcome the impact of fluorescence quenching caused by disordered aggregation of materials while also displaying characteristics of an optical waveguide [10,11,12,13,14,15]. To further study the intrinsic optical properties of such materials, an intramolecular charge transfer (ICT) compound with nitro-group acting as an electron acceptor and the nitrobenzothiadiazole unit as the electron donor was designed and synthesized. Supramolecular aggregates with well-defined morphologies have been constructed by simple self-assembly methods, and the effect of the nitro group on the photoelectric properties of the molecule was investigated. The nonlinear optical properties of this material were also explored and studied through the Z-scan method. It has been found that this material exhibits large third-order nonlinear absorption and refraction coefficients, which promises applications of the materials in the fields of nonlinear optics and optoelectronics.

## 2. Materials and Methods

### 2.1. Chemical Materials

Phenylboronic acid pinacol ester, 4-bromo-7-nitrobenzothiadiazole and Pd(PPh_3_)_4_ are the analytical pure reagents, K_2_CO_3_ and silica gel (200–300 mesh) are the chemical pure reagents and all of the above reagents were purchased from Sigma Aldrich (Shanghai, China) Trading Co., Ltd.

### 2.2. Experimental Equipment

The ^1^H, ^13^C nuclear magnetic resonance (^1^H NMR, 400 MHz; and ^13^C NMR, 100 MHz) spectra were collected with the Bruker ARX400 MHz spectrometer (Leipzig, Germany). The UV-Visible (UV-Vis) absorption spectrum was measured by Hitachi U-3010 UV-Visible spectrophotometer. The fluorescence spectrum was measured by Hitachi F-4500 fluorescence spectrometer (Chiyoda-ku, Tokyo, Japan). Nano-scale images of the structure were taken using a JEOL JSM 4800F field emission scanning electron microscope (SEM), atomic force microscope (AFM) and the Multimode Nanoscope controller IIIa (Veeco Inc., New York, United States). The Confocal fluorescence image was taken with the OLYMPUS FV1000-IX81 Confocal Laser Microscope (Tokyo, Japan). The high resolution mass spectrometry (HMRS) was measured by Varian 7.0T FTMS (Santa Clara, CA, USA).

### 2.3. Synthesis

**Scheme 1:**
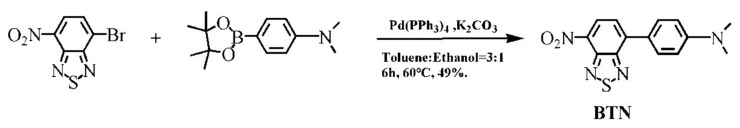
Synthesis route to the target compound **BTN.**

The target nitro compound, dimethyl-[4-(7-nitro-benzo[1,2,5]thiadiazol-4-yl)-phenyl]-amine (BTN), was obtained via the Suzuki coupling reaction between the 4-bromo-7-nitrobenzothiadiazole [16] and phenylboronic acid pinacol ester under the catalysis of Pd(PPh_3_)_4_ and K_2_CO_3_. The synthetic route is shown in Scheme 1, and the details are described below.

4-(*N*,*N*-dimethylamino) phenylborate pinacol ester (123 mg, 0.50 mmol) and 4-bromo-7-nitrobenzothiadiazole (129 mg, 0.50 mmol) were dissolved in a mixture of toluene (45 mL) and ethanol (15 mL). After 30 min of agitation at room temperature under nitrogen atmosphere, Pd(PPh_3_)_4_ (57 mg, 0.05 mmol) and K_2_CO_3_ (207 mg, 1.5 mmol) were added to the reaction mixture, which was stirred for 6 h at 60 °C. After the reaction was finished, the mixture was cooled to room temperature. H_2_O was added to quench the reaction, and then dichloromethane was used to extract (2 × 100 mL). The combined organic extracts were washed with water and then dried. The solvent was evaporated, the crude product was purified by silica gel column chromatography with dichloromethane as the eluent, to afford the product as the red solid (34.5 mg, 0.245 mmol) with a yield of 49%. ^1^H NMR (400 MHz, CDCl_3_): δ/ppm 3.10 (s, 6 H), 6.87 (d, *J* = 7.6 Hz, 2 H), 7.74 (d, *J* = 7.6 Hz, 1 H), 8.02 (d, *J* = 7.8 Hz, 2 H), 8.66 (d, *J* = 7.7 Hz, 1 H); ^13^C NMR (100 MHz, CDCl_3_): δ/ppm 154.4, 151.6, 147.8, 142.0, 136.5, 131.1, 128.7, 123.2, 122.9, 112.1, 40.2; HR GCT-MS: m/z: 323.0578 (M + Na^+^). Calc. Mass 323.0579. ^1^H NMR, ^13^C NMR and HMRS spectra were shown in Supplementary Material Figures S1–S3.

## 3. Results and Discussion

### 3.1. Self-Assembly

**Figure 1:**
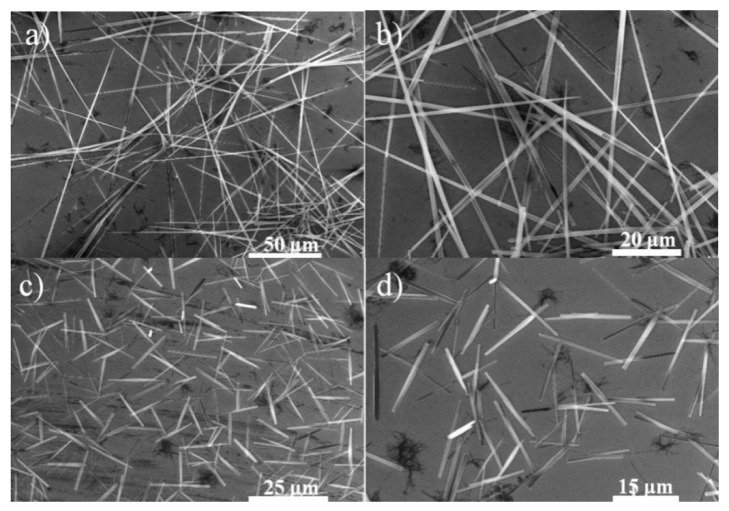
The self-assembled wire-like (**a**,**b**) and rod-like (**c**,**d**) aggregation structures of BTN.

Self-assembly is the process where molecules assemble into an ordered nanostructure through the interactions between molecules, such as electrostatic attraction, hydrogen bond and hydrophobic association [17,18]. This method is simple to operate and is suitable for small organic molecular compounds [19]. Large scale, uniformed micro- and nano- scale supramolecular aggregations can be obtained by the solution based self-assembly method with the absence of additional templates, catalysts and surfactant [20,21]. In this study, aggregated organic nano-scale structures were prepared by this method. Strong hydrogen bonds, π-π and dipole-dipole interactions among BTN molecules were observed by single crystal X-ray diffraction [22] (in Supplementary Materials). The saturated solution of BTN in tetrahydrofuran was slowly cooled down in an oil bath from 70 ℃ to room temperature, and stable one-dimensional wire-like structures were precipitated (Figure 1a,b). The widths of these structures range from hundreds of nanometers to a few microns and the lengths are of hundreds of microns.

The polarity of solvent has a great influence on the self-assembly of the compound. When tetrahydrofuran was changed to dichloromethane, one-dimensional rod-like structures were obtained during the same cooling process (Figure 1c,d). When the saturated refluxing solution of BTN was cooled at room temperature, self-assembly was expected to occur due to the limited solubility. The aggregates of BTN tend to stack along one axis due to the large dipole-dipole interactions and hydrogen bonds. The crystallization rate of BTN-1 in the tetrahydrofuran is higher than that in dichloromethane, leading to the different one-dimensional morphologies. Thus, the morphologies of BTN could be easily controlled through adjusting the different solvents. Powder X-Ray Diffraction (XRD) data indicates that these wire-like and rod-like aggregation structures are highly crystalline (Figure S4). The AFM image shows that the surfaces of these aggregation structures are very uniform (Figure S5).

### 3.2. Crystal Structure

**Figure 2:**
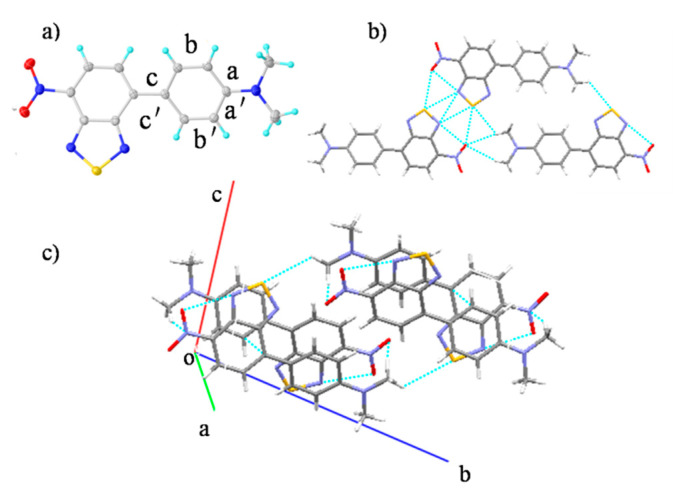
(**a**) Ortep view of BTN with thermal ellipsoids drawn at 50% probability level. (**b**) Front view of molecules with short interactions. (**c**) Crystal packing structure.

Single crystals of BTN were obtained by the two-phase solvent diffusion process with tetrahydrofuran as the good solvent and hexane as the poor solvent. Single crystal X-ray diffraction analysis was carried out to understand the crystal structures (Table 1). The results show that the single crystal belongs to a monoclinic P2_1_/n space group. The whole molecule presents a distorted structure, and the angle between the benzothiadiazole unit and the adjacent benzene ring is 26.6° (Figure 2a). As shown in the stacking diagram (Figure 2c), a unit cell of BTN single crystal contains four molecules, two pairs of which are stacked in opposite dipole directions. When viewed along the crystallographic *b*-axis, the molecular accumulation presents two kinds of antiparallel stacking. The crystal stacking is driven by the short heteroatomic forces such as S_1_···N_2_ (3.02 Å), N_2_···N_2_ (2.97 Å) between adjacent 1,2,5-thiadiazole heterocycles (Figure 2b). There are also rich hydrogen bonds between molecules, such as S_1_···H-C (2.93 Å) between the heteroatom S and the O···H-C (2.67 Å, 2.58 Å) formed by the oxygen atom on nitro group with two hydrogen atoms from two adjacent methyl groups separately. Combined with these forces, the molecules exhibit a head-tail-head-tail stacking structure [23].

The change of the bond length on the *N*,*N*-dimethyl benzene ring can reflect the degree of ICT between the acceptor and the donor. This can be shown by the value of δ_r_ of benzoquinone [24]. δ_r_ is equal to 0 for benzene (see Figure 2a for the definition of the bonds of *a*, *a*′, *b*, *b*′, *c*, *c*′). The δ_r_ of BTN is 0.029, which indicates that BTN shows a very effective intramolecular charge transfer.

(1) $$\delta_{r}=[(a+a\prime )/2-(b+b\prime )/2]+[(c+c\prime )-(b+b\prime )]/2$$

### 3.3. Theoretical Analysis of Compounds

**Figure 3:**
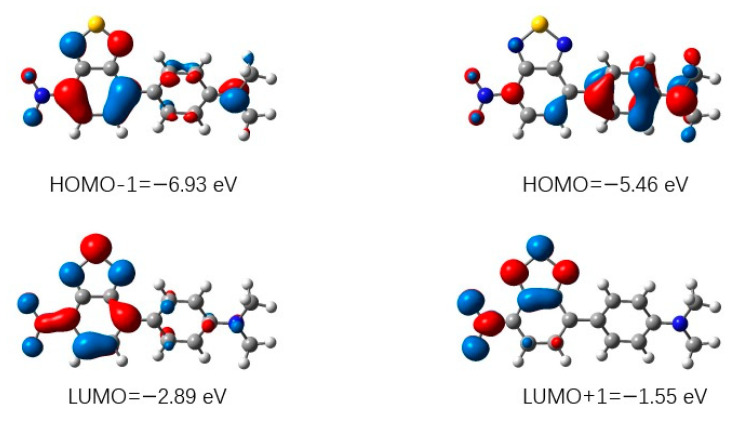
Calculated frontier molecular orbitals of BTN.

According to the molecular orbital theory, the highest occupied molecular orbitals (HOMO) and the lowest occupied molecular orbitals (LUMO) are the main orbitals that contribute to the chemical stability of molecules and have an important influence on the optoelectronic activity of compounds [25]. In order to further understand the spatial configuration and electron cloud distribution of the molecule, density functional theory (DFT) was applied to optimize the molecular configuration and evaluate the electron cloud distribution of BTN in vacuum. The calculation was run by Gaussian 09 software package with B3LYP and 6-31G* as the base group [26,27]. As can be seen from Figure 3, the electron cloud on the HOMO orbital is more evenly distributed on the conjugated surface of *N*,*N*-dimethyl-benzene ring, while the electron cloud on the LUMO orbital is mainly located on the benzothiadiazole ring and the nitro group, with the electron cloud density on the benzene ring significantly reduced. The HOMO to LUMO charge transfer is mainly attributed to the n-π transition from donor to acceptor [28].

### 3.4. Electrochemical Properties

**Figure 4:**
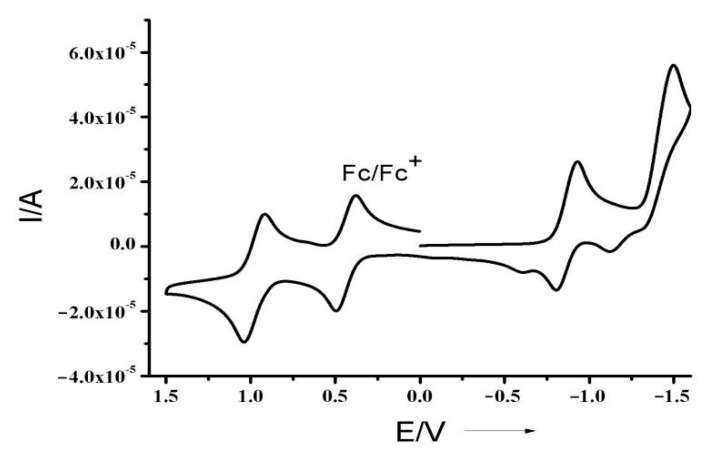
Cyclic voltammetry of BTN. The scanning speed is 0.1 V/s, and the scanning direction is from 0 to positive, then back to negative.

The electrochemical properties of BTN were studied by cyclic voltammetry. The experiment was carried out in dichloromethane with oxygen removed beforehand. The glassy-carbon electrode was used as the working electrode, platinum wire as the opposite electrode and saturated calomel electrode as the reference electrode. The solution contained 0.1 M tetra-n-butylammonium hexafluorophosphate as the supporting electrolyte and ferrocene as the internal standard. It can be seen from the cyclic voltammetry that the compound has a reversible oxidation peak (0.98 V) in the oxidation region (Figure 4), which can be attributed to the oxidation of *N*,*N*-dimethyl units. In the reducibility area, a reversible reduction peak and an irreversible reduction peak are present. The reversible reduction peak (−0.85 V) can be attributed to the reduction of the receptor nitro group. The irreversible reduction peak (−1.40 V) can be attributed to the reduction of benzothiadiazole unit.

### 3.5. Linear Optical Properties

**Figure 5:**
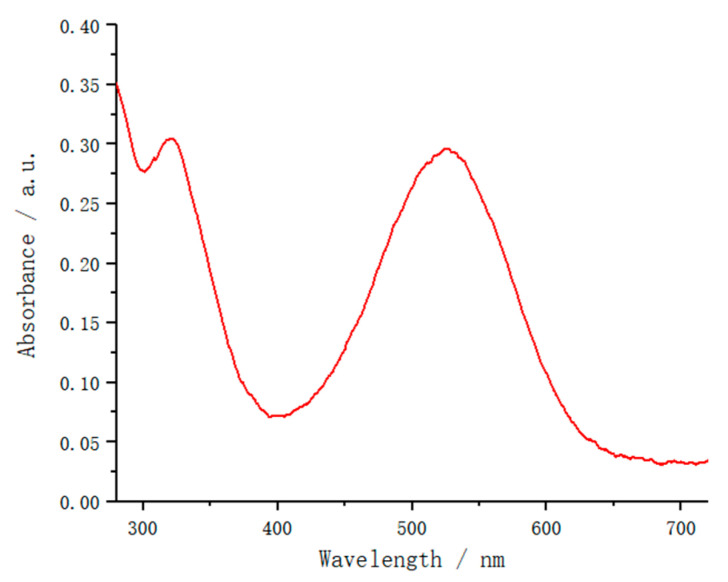
The UV-Vis absorption diagram of BTN solution in methylene chloride.

The UV-Vis absorption spectrum of BTN as measured from its solution in methylene chloride solution suggests a narrow absorption band at near 320 nm (Figure 5), which is caused by the π-π* transition of benzothiadiazole units [29]. Meanwhile, there is a strong ICT peak centered at 514 nm in the visible region. The absorption of this wide peak is mainly due to the n→π* transition induced by the ICT process [30].

**Figure 6:**
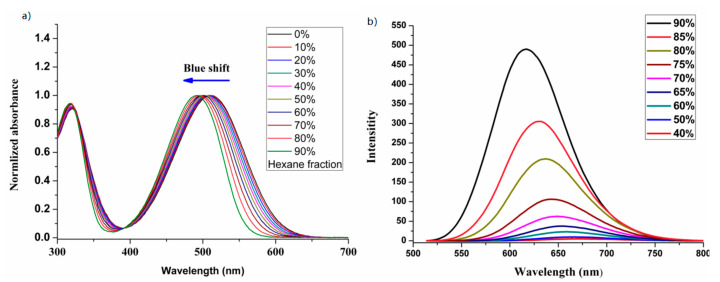
UV-Vis absorption spectra (**a**) and fluorescence emission spectra (**b**) of BTN with different ratios of tetrahydrofuran and *n*-hexane. Concentration: 2.0 × 10^−5^ M.

It is widely accepted that nitro-compounds such as BTN hardly fluoresce in the solution due to the strong electron absorption of nitro group and the effect of orbital spin coupling [31,32]. Nevertheless, we studied the luminescence behavior of BTN in both the solution and aggregation states. The results showed that the solution of BTN in pure tetrahydrofuran did not emit light when excited at its maximum absorption wavelength of 514 nm. However, at a certain concentration (total concentration 2.0 × 10^−5^ M), the luminescence of tetrahydrofuran solution was significantly changed when a certain proportion of *n*-hexane was introduced. As the proportion of *n*-hexane gradually increases, the luminescence intensity of the solution gradually increases accordingly, and the maximum emission wavelength has a significant blue shift (Figure 6a). The maximum fluorescence emission peaks range from 672 to 617 nm. However, the maximum absorption wavelength of the solution was not as sensitive to the change of the fractions of mixture solution. When the percentage of hexane was increased from 0% to 70%, 80% and 90%, the ICT band red-shifted gradually from 514 to 496, 494 and 492 nm (Figure 6b, Table S1), showing the AIE effect [7,33]. BTN in good solution is polarized and highly distorted, featuring the nonradiative decay progress. However, if it is aggregated from the dilute solution, the intramolecular charge transfer will be eliminated, and the emissions will be recovered.

The luminescence of the aggregation state of BTN was also observed by fluorescence confocal microscope (Figure S6). The compound was demonstrated to be a good luminescent material showing yellowish green fluorescence in the aggregation state. This property overcame the fluorescence quenching phenomenon caused by aggregation in the solid state of traditional luminescent emission materials, which was a typical AIE phenomenon. There are several widely accepted mechanisms, such as the RIM, JAF and excimer mechanisms. However, the AIE properties of the ICT compounds with nitro groups are more related to the change of molecular structure and state [11]. In nitro compounds with a certain conjugated system, the fluorescence is quenched due to the strong charge transfer in the excited state. When the molecules are in the aggregation state, due to the C-H···O hydrogen bonds between nitro groups, benzene rings and methyl groups, and the interaction between heteroatoms, some electrons are provided to offset the electron absorption ability of nitro groups, and the π-conjugated system is released and the fluorescence is displayed and restored.

### 3.6. Nonlinear Optical Properties

The nonlinear optical properties of BTN were studied by a Z-scan technique in its dichloromethane solution [34]. We first used CS_2_ as the standard sample to calibrate the system. CS_2_ was placed in the quartz sample pool, and the closed-aperture curve was obtained [32]. The nonlinear refractive index coefficient of CS_2_ was calculated to be 5.7 × 10^−17^ m^2^/W, which is very close to the reported value in the literature [35]. The nonlinear response of the quartz substrate and the sample cell is very weak, and their influence on the experimental results can be ignored.

**Figure 7:**
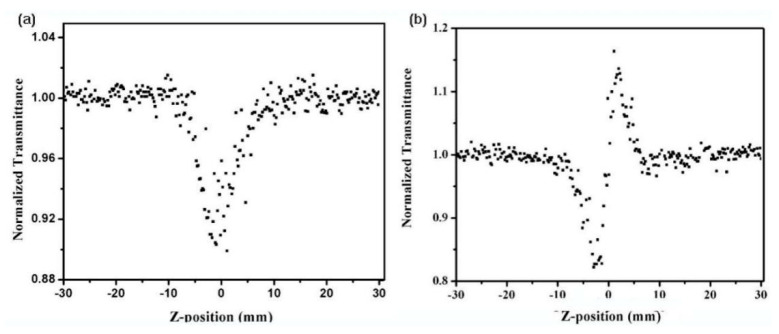
(**a**) The open-aperture Z-scan curve of BTN in 2 mm thick quartz sample cell. (**b**) The closed-aperture Z-scan curve of BTN in 2 mm thick quartz sample cell.

We first measured the nonlinear absorption of the sample BTN under the condition of open-aperture, and the obtained curve is shown in Figure 7. It can be seen that the energy transmittance of the sample at the focus is only about 0.9 of the initial energy, which indicates that BTN exhibits anti-saturation absorption behavior.

The nonlinear absorption data of samples can be expressed by Equation (2):

(2) $$\begin{array}{l} T(z)=\frac{1}{\sqrt{\pi q(z)}}\int_{-\infty }^{\infty }\ln[1+q(z)]e^{-\tau^{2}}d\tau \\ q(z)=\alpha_{2}^{eff}I(z)\frac{1-e^{-\alpha_{{}_{0}}L}}{\alpha_{0}} \end{array}$$

In the formula above, ***α*_0_** and ***α*_2_** are linear and nonlinear absorption coefficient, ***τ*** is time, ***L*** is the thickness of the sample. Transmittance (***T***) is a function of sample position ***Z*** (***Z*** = 0 at the focus). By fitting the data with the five-level model [36], which takes into account the dynamic thermal effect resulting from the transient excited-state absorption. The effective nonlinear absorption coefficient ***α*_2_** of BTN solution can be calculated to be 2.9 × 10^−11^ m/W.

The data points shown in black squares in Figure 7 are curves of nonlinear refraction of BTN solution under closed-aperture condition. The nonlinear refraction effect of samples is obtained by removing the normalized closed-aperture Z-scan data from the normalized open-aperture Z-scan data. This is a curve pattern of a valley followed by a peak, which indicates that the solution has a self-focusing characteristic. The third-order nonlinear refractive index coefficient n_2_ can be given by the valley peak difference (Δ*T*_*v*−*p*_) in the curve and combined with Formula (3) to give [37].

(3) $$n_{2}=\frac{\lambda \alpha_{0}}{0.812\pi I(1-e^{-\alpha_{0}L})}\Delta T_{v-p}$$

In formula (3), the difference between the peak and valley values of **Δ*T_v*−*p_*** normalized transmittance, ***I*** is the focus intensity, and ***λ*** is the wavelength of the laser. Through calculation, it can be estimated that the **n_2_** value of the nonlinear refractive index coefficient of BTN is 3.4 × 10^−18^ m^2^/W. These results show that BTN has high third-order nonlinear absorption and nonlinear refraction coefficients, which promise great potential of these material for applications in well-defined nonlinear optical materials.

## 4. Conclusions

In this article, starting from the principle that the photoelectric performance of ICT molecules can be regulated by adjusting the efficiency of charge transfer, we have synthesized an ICT compound BTN with rich self-assembly and optoelectronic properties. The ICT compound has been constructed into well-defined supramolecular aggregates with specific morphologies by using intermolecular non-covalent weak interactions such as hydrogen bonds, π-π, dipole-dipole and Van der Waals forces as the driving forces. The single crystal analysis shows the strong ICT characteristic of this ICT compound. The aggregation structures of micro-wire and micro-rod were obtained by solvent saturation precipitation. This compound has no fluorescence in the solution state, but show strong fluorescence in aggregation state, which overcomes the fluorescence quenching phenomenon caused by aggregation and has potential applications in organic light-emitting diode and other related fields. The compound also exhibits strong third-order nonlinear absorption and refraction coefficient, which promises potential applications in nonlinear photonic devices.

## Figures and Tables

**Table 1:** Crystal structure data of BTN.

Formula	C_14_H_12_N_4_O_2_S
CCDC number	1977053
Mr	300.34
crystal size (mm^3^)	0.40 × 0.20 × 0.07
crystal system	Monoclinic
space group	P2_1_/n
a (Å)	13.053(3)
b (Å)	6.9678(14)
c (Å)	14.799(3)
α(deg)	90
β (deg)	106.85(3)
γ (deg)	90
V (Å^3^)	1288.2(4)
Z	4
F(000)	624
calcd (mg m^−3^)	1.549
(mm^−1^)	0.262
range (deg)	1.83 to 27.52
R1 [I > 2σ(I)]	0.0481
wR2 [I > 2σ(I)]	0.1163
goodness of fit	1.150

## Data Availability

Data sharing is not applicable to this article.

## Supplementary Materials

The following are available online at https://www.mdpi.com/article/10.3390/ma14081909/s1, Figure S1: ^1^H NMR spectrum of BTN, Figure S2: ^13^C NMR spectrum of BTN, Figure S3: HRMS pattern of BTN, Figure S4: XRD patterns of BTN crystals with two structures and simulated XRD patterns of single crystal XRD data from rod-like single crystals, Figure S5: The AFM image of a typical single micronwire of BTN, Figure S6: The fluorescence confocal microscope of BTN nanowires, Table S1: The maximum absorption and fluorescence emission peak of BTN in different ratios of hexane and tetrahydrofuran.
