# Supplementary material for: Aggregation Induced Emission and Nonlinear Optical Properties of an Intramolecular Charge-Transfer Compound
Source: Materials (Basel). 2021 Apr 11;14(8):1909. doi: 10.3390/ma14081909 (PMC8069476; doi:10.3390/ma14081909)
Supplement: Supplementary file 1 [file materials-14-01909-s001.pdf]

materials

MDPI

Supplementary Materials

Aggregation Induced Emission and Nonlinear Optical Properties of an Intramolecular Charge-Transfer Compound

Songhua Chen [1], Rui Luo [2], Xinyue Li [2], Meiyun He [1], Shanshan Fu [1] and Jialiang Xu [2,*]

[1] College of Chemistry and Material Science, Longyan University, Longyan 364012, China; songhua@iccas.ac.cn (S.C.); meiyunhmy@163.com (M.H.); shanshanFu33@163.com (S.F.)
[2] School of Materials Science and Engineering, National Institute for Advanced Materials, Nankai University, Tongyan Road 38, Tianjin 300350, China; 1813791@mail.nankai.edu.cn (R.L.); xinyueli@yeah.net (X.L.)
* Correspondence: jialiang.xu@nankai.edu.cn

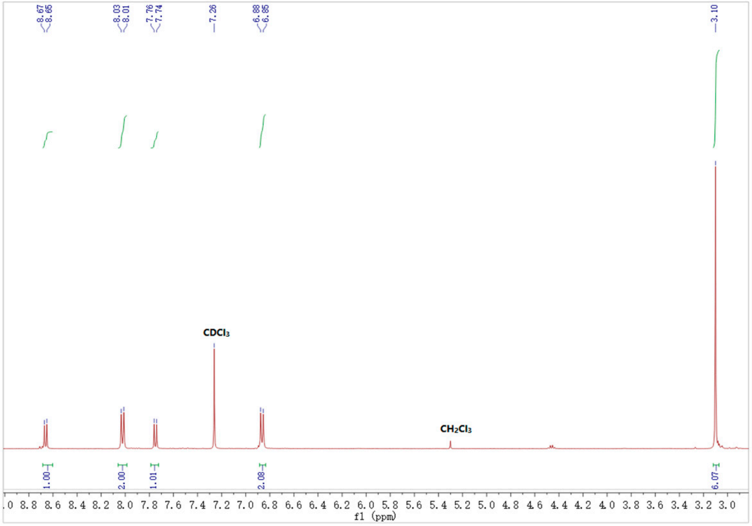

Figure S1. ^{1}H NMR spectrum of **BTN**.

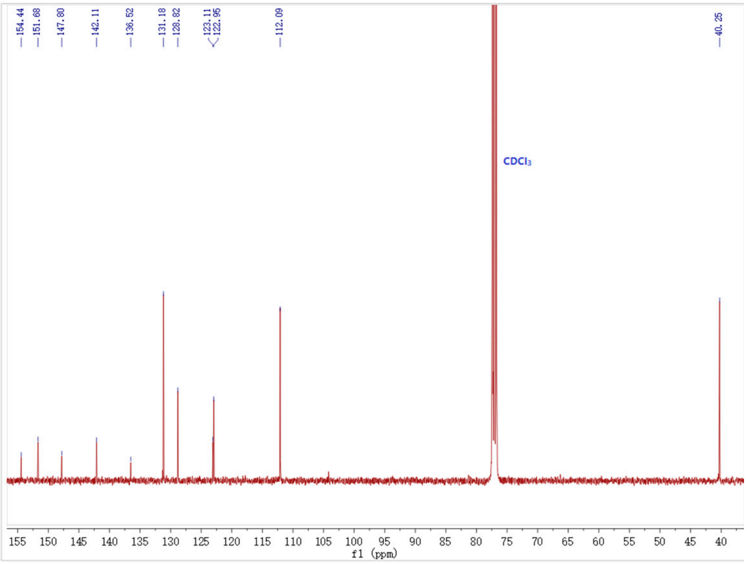

Figure S2. ^{13}C NMR spectrum of **BTN**.

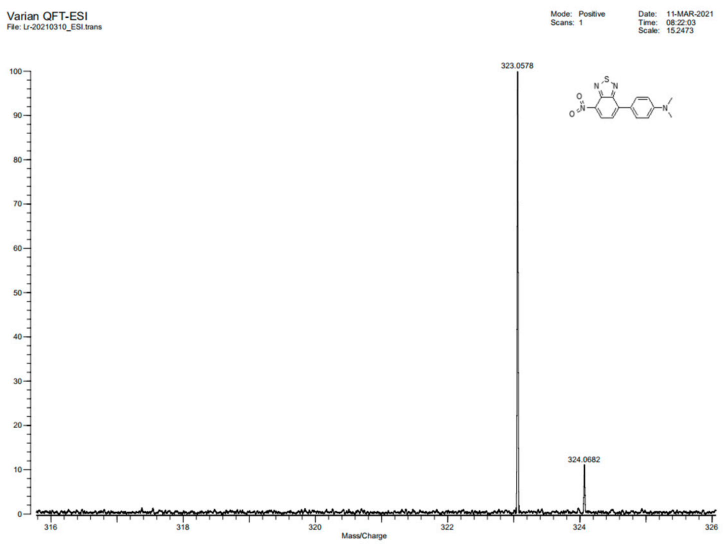

Figure S3. HRMS pattern of **BTN**.

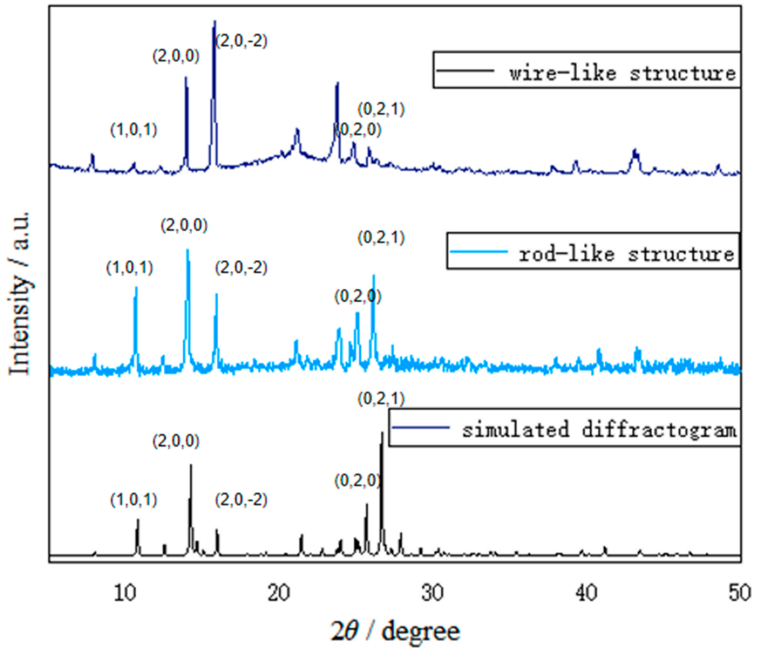

Figure S4. XRD patterns of BTN crystals with two structures and simulated XRD patterns of single crystal XRD data from rod-like single crystals.

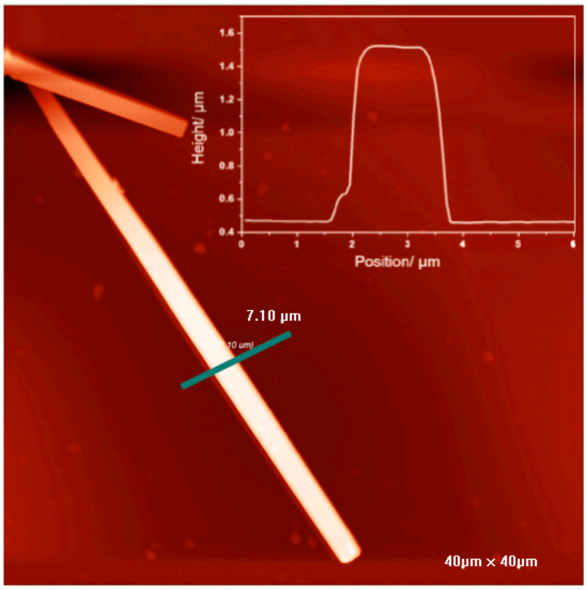

Figure S5. The AFM image of a typical single micronwire of **BTN**.

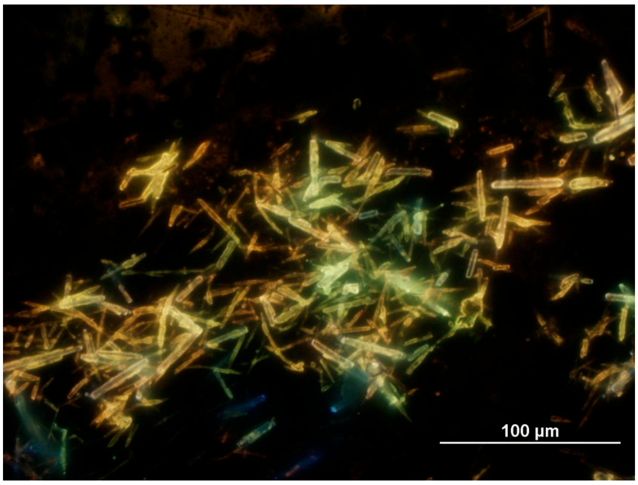

Figure S6. The fluorescence confocal microscope of **BTN** nanowires.

Table S1. The maximum absorption and fluorescence emission peak of **BTN** in different ratios of hexane and tetrahydrofuran.

Hexane Fraction(%)	0	10	20	30	40	50	60	65	70	75	80	85	90
λ_{max}^{abs}	514	512	508	504	504	502	500	498	496	496	494	494	492
λ_{max}^{em}	-	-	-	-	672	666	657	655	649	643	638	631	617

© 2021 by the authors. Licensee MDPI, Basel, Switzerland. This article is an open access article distributed under the terms and conditions of the Creative Commons Attribution (CC BY) license (http://creativecommons.org/licenses/by/4.0/).
